# Buffalo Medical and Surgical Association

**Published:** 1884-04

**Authors:** Wm. D. Granger

**Affiliations:** Acting Secretary


### Buffalo Medical and Surgical Association.

*Regular Meeting, March 4th, 1884.*

President, Dr. J. C. Cronyn, in the Chair.

Present—Drs. Rochester, Hopkins, Strong, Mann, Brecht, Mynter, Van Peyma, Coakley, O'Brien, Bartlett, Park, Putnam, Frank, Long, Parmenter, Granger, and, by invitation, Drs. Howell and Park.

Essay by Dr. Roswell Park. Subject: "Selected Topics in the Surgery of the Nervous System."

The subject was divided into three parts, of which the first was on nerve suture. The literature of the subject was first referred to, as well as some of the early experiments. The matter had now been before the profession in some shape or other for seventeen years, and various writers had reported cases, until now, as far as he could ascertain, some thirty odd cases were on record, nearly all of them successful. To these the Doctor desired to add two of his own. The first, a boy of thirteen, upon whom he was obliged to make an extensive operation for tuberculous caries of the bony pelvis and its contents. A large flap composed of the entire buttock was laid off and the sciatic nerve exposed. The flap was held aside with a strong retractor, when suddenly the patient, having been inadvertently allowed to partially recover from his deep anæsthesia, gave a wrench and turned partially over; by this procedure the retractor was hooked under the nerve and tore it completely through. The operation was completed as if nothing had happened, the major portion of the ischium resected, sinuses in the pelvis scraped out, and at its close the nerve ends were reunited by two catgut sutures. There was complete paralysis of sensation and motion for a week in those parts supplied by the nerve; but at the end of the second week sensibility was very nearly perfect and motility was completely restored.

The second case was that of a lad who cut some of the extensor tendons of the hand as well as the radial vessels and nerve. After two weeks the tendons and nerve were united by suture, with return of sensation and almost perfect restoration of function.

After the relation of his cases the Doctor gave some general directions as to the precautions to be observed and the carrying out of those details so essential to success; he also referred to the work of experimental physiologists in this direction, to transplantation of nerves, and to the fact that neither tetanus nor other untoward results of this sort of surgery had been known to occur.

The second part of the paper dealt with elongation of nerves. A brief history of the operation was first given, and then reference was made to its applicability in certain classes of cases, after which the reader reported five cases of nerve stretching. The first was for sciatica, with subsequent recovery; the second for tetanus, the brachial plexus being stretched, with amelioration of symptoms. The other three were done purely as physiological experiments, with the full knowledge and consent of the patients, for obscure nervous affections which had resisted all other internal and external treatment. In one of them there was slight improvement; in none did any evil result. After reporting the cases, the Doctor commented suitably on each one, and quoted cases where elongation had cured paralysis, disorders of sensation, and had even restored sensation in anæsthetic leprosy.

The last portion of the paper was devoted to the exploration of the brain by the hollow needle. The reader made the statement that he was not aware of any special literature bearing on the subject, but would report three instances in which he had made exploration of the hemispheres by means of the long needle of the hypodermic syringe in search after pus, of course after removal of a button of bone by the trephine. He brought forward his own cases to show how simple and harmless the procedure was, even if nothing but negative evidence was elicited.

The whole paper was an exceedingly practical one, and contained many references to the literature of the first two topics discussed. It will probably be published hereafter in full.

*Discussion*—Dr. Rochester expressed his interest in the subject, and his pleasure in the paper presented. He related a case of tetanus in a married woman. The injury was a nail in the foot, followed by pain, and in four days slight symptoms of tetanus. Patient placed in bed; wound searched for foreign body. Bromide, gr. 30, morph., gr. ½, given every four hours. The Doctor remarked that he knew the two remedies were considered incompatible, but they were the only ones he had known to do any good in this disease. The patient got better, but against orders left the bed, and the inflammation returned, with a return of the tetanic symptoms. Dr. Mynter was called. He enlarged the wound, again searched for foreign body, and severed the nerve. Hypodermic injections of physostigma were given without controlling the disease. Patient died in five days after second attack.

Dr. Strong asked about the transplantation of nerve, and also how much force could be used.

Dr. Mynter expressed much interest, and also praised the frankness of Dr. Park in criticising his own cases, and being willing to report a case in which there was an error in diagnosis, saying it was too often the habit to speak of one's successes and forget any mistake. He reported a case of laceration of the hand where he united tendons, but searched for and could not find the nerves. He used antiseptic measures, and placed the hand in a splint. There was good recovery with slow return of movement, and slower but very fair return of sensation.

He favored nerve stretching for neuralgia, but never saw a case of sciatica bad enough to operate upon. He had tried one case by hyperflexion of the knee, under chloroform, with good result Also reported a case of trephining for epilepsy. A man, at thirteen years of age had a fracture of the skull. There was depression, and when the spot was pressed upon showed a very sensitive point. Increasing severity of the fits, and deepening dementia, decided him to operate. Several buttons of skull were removed, and a center splinter that extended into the brain. Acute mania followed, and he died in four days. Autopsy showed an abscess and atrophy of the side of the brain and dilatation of ventricles.

Dr. Coakley spoke of the reproduction of nerve tissue, and quoted Dalton as authority that nerves, like muscles and bones, were reproduced.

He asked why Dr. Park taught that gun-shot wounds should not be freely explored. He was always in the habit of using the finger as a probe.

Dr. Putnam spoke of section and stretching of nerves. He quoted Anstie as denouncing section of nerves, and says it gives no success except in neuralgia of fifth pair, and says section of others should not be made. He also claimed that experiments upon the cadaver showed that the sciatic nerve could be stretched an inch by hyperflexion and other manipulations. Of the value of nerve stretching, he said that surgeons advise and physicians decry it. He also claimed, still quoting from Anstie, that a careful analysis of cases showed few recoveries but many cases temporarily benefited, and claimed the best results after all other treatment failed was found in the continued current.

Dr. Frank saw two cases of nerve stretching without benefit. He spoke of a case reported a year ago of circumcision for convulsions, with entire relief; and also of another case reported cured in a journal.

Dr. Van Peyma asked for information in regard to nerve reuniting.

Dr. Cronyn spoke of the importance of many details of the paper that might seem unimportant to many; yet they were of great practical value. It was well to remember that in nerve suture there was one particular way to introduce the needle so as to injure the fewest number of nerve fibers. The whole subject is on trial, and the discrimination of cases is yet to be made. He spoke of a case of fracture of the right occipital bone. He cleaned wound, and dressed it; but four days after, while straining at stool, hernia of brain took place. Convulsions followed, and chloroform giving no relief, he ligatured the mass of brain, as large as a pullet's egg, and cut it off. The convulsions ceased, child recovered and is now living, a bright person, having gone through the public schools. He spoke of another case, in which a child received a gun-shot wound. The bullet passed through left orbital plate. After recovery the child had forgotten how to read, and did not know one letter from another, but his arithmetic was improved. He learned over again.

Nine years later the child was killed by a blow on the other side of the head, and bullet was found encysted.

Dr. Park, replying to questions and criticisms, said that just how much stretching a healthy nerve will bear is uncertain. One experiment showed the sciatic would sustain a weight of 80 kilogrammes, another 75 kilogrammes, and a third 180 pounds. His rule was to lift the limb entirely by the nerve and pull both ways. Pulling from above gives irregular nerve action. Newer teaching forbids doing anything to gun-shot wounds until ready to operate and everything is at hand. In regard to difference of opinion between surgeons and physicians, he thought failure to obtain results was often due to timidity, and therefore failure to properly stretch the nerve. The sensitive fibres were first paralyzed, and greater force is required to affect the motor nerves. It is difficult to say how nerves regenerate. Lower animals more easily reproduce lost tissue, and the higher the tissue the more difficult the reproduction. As much as two inches will fill in. First, there is cicatricial tissue, then vessels are thrown out, and finally nerve fibres meet. In sutures of freshly cut nerves there is first degeneration of the lower end, and then gradual reproduction of tissue.

*Voluntary Communications*—Dr. Park presented a specimen of trichina, a microscopic preparation.

*Reports of Prevailing Diseases*—Dr. Rochester reported three fatal cases of diphtheria in one family, and also noted the fact that the three other children escaped by being removed from the house. He thought they contracted the disease at school.

Dr. Hopkins reported bronchial catarrh and pneumonia.

Dr. Bartlett reported some diphtheria, some whooping cough, measles and scarlet fever, with little rash.

Dr. Frank reported measles and mumps.

Dr. O'Brien reported measles and bronchitis.

Dr. Van Peyma reported measles, scarlet fever and broncho-pneumonia.

Dr. Coakley reported rheumatism.

Dr. Cronyn reported sore throat without rash, and calls it herpetic form of tonsillitis.

It was announced by the President that Dr. M. D. Mann would read essay at the next meeting, in April. Subject: "Axis Traction Forceps, with Exhibition of Instruments."

WM. D. GRANGER, *Acting Secretary.*

